# Application of AULA Risk Assessment Tool by Comparison with Other Ergonomic Risk Assessment Tools

**DOI:** 10.3390/ijerph17186479

**Published:** 2020-09-05

**Authors:** Kyeong-Hee Choi, Dae-Min Kim, Min-Uk Cho, Chae-Won Park, Seoung-Yeon Kim, Min-Jung Kim, Yong-Ku Kong

**Affiliations:** 1 Department of Industrial Engineering, Sungkyunkwan University, Suwon 16419, Korea; kyunghe7@naver.com (K.-H.C.); crayonmm@naver.com (M.-U.C.); cwrachel@naver.com (C.-W.P.); kimsy9035@naver.com (S.-Y.K.); xlsk1013@naver.com (M.-J.K.); 2 Division of ICT Convergence Engineering, Dongseo University, Busan 47011, Korea; dmkim@gdsu.dongseo.ac.kr

**Keywords:** ergonomic risk assessment tools, hit rate analysis, quadratic weighted kappa analysis, AULA, REBA, RULA, OWAS

## Abstract

Agricultural upper limb assessment (AULA), which was developed for evaluating upper limb body postures, was compared with the existing assessment tools such as rapid upper limb assessment (RULA), rapid entire body assessment (REBA), and ovako working posture analysis system (OWAS) based on the results of experts’ assessments of 196 farm tasks in this study. The expert group consisted of ergonomists, industrial medicine experts, and agricultural experts. As a result of the hit rate analysis, the hit rate (average: 48.6%) of AULA was significantly higher than those of the other assessment tools (RULA: 33.3%, REBA: 30.1%, and OWAS: 34.4%). The quadratic weighted kappa analysis also showed that the kappa value (0.718) of AULA was significantly higher than those of the other assessment tools (0.599, 0.578, and 0.538 for RULA, REBA, and OWAS, respectively). Based on the results, AULA showed a better agreement with expert evaluation results than other evaluation tools. In general, other assessment tools tended to underestimate the risk of upper limb posture in this study. AULA would be an appropriate evaluation tool to assess the risk of various upper limb postures.

## 1. Introduction

The possibility of work-related musculoskeletal disorders (WMSDs), which are accompanied by pain in muscles, tendons, and nerves, increases due to repetitive activities and awkward working postures [1]. According to statistical reports, the population growth rate of the elderly is faster than the overall population growth rate, and this phenomenon is more pronounced in rural areas [2,3]. The aging of the rural regions and the decrease in the farming population per household has led to a severe reduction in the labor force and an increase in labor intensity, causing serious WMSDs problems for farmworkers [4,5]. The prevalence of WMSDs, particularly in the trunk, shoulder, and hand/wrist, is very high in Asian farmers [6,7,8,9]. The prevalence (61.5%) of WMSDs among farmworkers, forestry workers, and fishers was 2.5 times higher than that (25.1%) of workers in other fields [10]. Therefore, it is important to determine the work-related risk factors to reduce WMSDs in the agriculture environment [11]. 

Few studies were conducted on the development of ergonomic assessment tools that accurately assess the postures of agricultural workers, despite the increased risks of WMSD in agricultural workers due to the aging of the rural population. The current ergonomic risk assessment checklists provide efficient assessment tools that enable assessing the risks of tasks by quickly and conveniently evaluating the agricultural tasks. Rapid upper limb assessment (RULA) [12], rapid entire body assessment (REBA) [13], and the ovako working posture analysis system (OWAS) [14] are widely used as ergonomic risk assessment tools to analyze various tasks in the industrial sector. However, each assessment tool has a different purpose and background of development, so the risk assessment is diverse depending on the type of task being analyzed [15,16,17,18,19].

According to the comparison of risk assessments for various tasks using OWAS, RULA, and REBA, OWAS and REBA generally tend to underestimate the risk of working posture compared to RULA [20,21,22,23,24,25]. Another study of subjective discomfort and the results of risk assessment tools showed that the REBA score showed a high correlation with the subjective discomfort of workers compared to OWAS and RULA [15]. Thus, it is reported that the existing ergonomic risk assessment tools were developed to assess the risks of frequently occurring working postures at industrial sites, so there are some limitations to applying them to the assessment of agricultural working postures [26]. 

Agricultural lower limb assessment (ALLA) for evaluating the risks of specialized lower extremity postures in agricultural tasks was developed by Kong, Han, and Kim (2010) [4]. They also compared ALLA with conventional ergonomic risk assessment tools (RULA, OWAS, and REBA) to verify that ALLA is a better assessment tool for various lower limb working postures in agricultural tasks [26]. The agricultural upper limb assessment (AULA) tool to evaluate the risks of upper extremity postures in agricultural tasks was also developed by Kong, Lee, Lee, Han, and Kim (2011) [27]. They developed the AULA ergonomic checklist based on an analysis of 14 upper limb postures that frequently occur in agricultural tasks. However, the developed AULA requires validation of an evaluation tool for agricultural tasks by comparing posture evaluations with existing assessment tools.

Therefore, the purpose of this study was to verify AULA developed for agricultural workers by comparing it with existing evaluation tools (RULA, REBA, and OWAS) for various types of actual agricultural working postures.

## 2. Materials and Methods 

### 2.1. Selection of Working Postures

**Figure 1:**
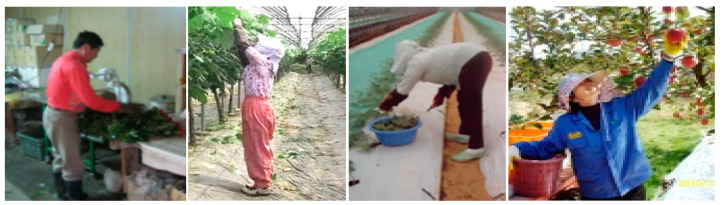
Examples of selected working postures.

To verify the evaluation of upper limb assessment tools (AULA, RULA, REBA, and OWAS), 196 working postures were selected from various crops according to the height of crops (Figure 1). A total of 10 crops were selected and consisted of four crops at under knee height, two crops at waist height, and four crops at shoulder height, as presented in Table 1.

Three researchers who completed the training on the four assessment tools (AULA, RULA, REBA, OWAS) evaluated the selected working postures by using four assessment tools. For a more accurate evaluation, all working pictures were printed, and the angle of each body segment was measured using a protractor. Based on the measured angle value, the four scores were calculated for each posture, and cross-checked between researchers to obtain more accurate data.

### 2.2. Evaluation of Ergonomic Experts

In this study, experts evaluated agricultural working postures for the risk of upper extremities. A total of 16 experts with more than 10 years of experience participated in this study. The expert group consisted of 6 experts in the ergonomics field, 6 experts in the industrial health field, and 4 experts in an agricultural area. Each expert assessed the risk level of the upper extremity of agricultural working posture on a 10-point scale (1: very safe posture, 10: very risky posture). According to the mean score measured by the experts, the risk level was classified into four levels: 1: low (1.00–3.25), 2: medium (3.25–5.50), 3: high (5.50–7.25), and 4: very high (7.25–10.00). We evaluated each assessment tool’s suitability with how well-matched each assessment tool’s results compared with the experts’ assessments.

### 2.3. Evaluation of Ergonomic Assessment Tools

**Figure 2:**
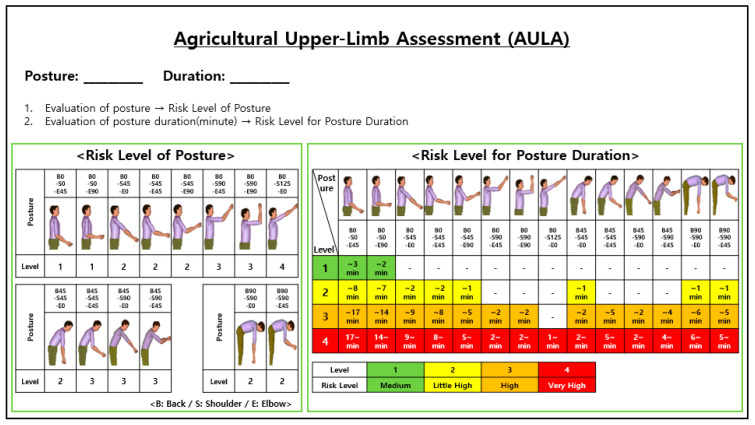
Agricultural upper limb assessment (AULA).

The ergonomic risk assessment tools (AULA, OWAS, RULA, and REBA) for upper-limb postures in selected working postures were evaluated in this study. As shown in Figure 2, the AULA assesses 14 working postures frequently occurring in agricultural work [26,27] and has four risk levels: moderate (lowest level), little high, high, and very high (highest level). The assessment of the upper-limb risk level was performed in RULA, REBA, and OWAS, assuming the same evaluation posture of the AULA: standing posture. 

In this study, all the evaluation tools were intended to assess the risk of agricultural work at four risk levels. Three assessment tools (AULA, RULA, OWAS) except REBA already had four risk levels, whereas REBA had five risk levels: negligible risk, low risk, intermediate risk, high risk, and very high risk. Thus, four risk levels were reconstructed to combine ’negligible risk’ and ’low risk’ to enable comparison with other assessment tools.

### 2.4. Statistical Analysis

To assess whether the AULA, RULA, REBA, and OWAS assessments matched experts’ assessments, hit rate analysis, quadratic weighted kappa analysis, and one-way ANOVA were performed.

For the hit rate analysis, experts first classified 196 working postures as risk levels 1, 2, 3, and 4 for low, medium, high, and very high-risk working postures, respectively. The working postures were also classified at four risk levels using the RULA, REBA, OWAS, and AULA assessment tools, as mentioned in Section 2.3; the hit rate of expert and each assessment tool was calculated. The quadratic weighted kappa analysis was also evaluated based on the results of experts and assessment tools. The kappa value from the analysis ranges between 0 and 1. The analysis was conducted according to the kappa value analysis criteria shown in Table 2 [28]. One-way ANOVA was conducted to identify the differences between the results of the assessment tools and experts’ assessment for all the risk groups (risk levels 1–4) in this study.

## 3. Results

### 3.1. Hit Rate Analysis

Table 3 shows the hit rate analysis results to investigate how well experts’ risk level assessment and the risk level assessment of the assessment tool matched for 196 farm tasks. The hit rate between experts and AULA assessment was 66.7% for risk level 1, and risk levels of 2, 3, and 4 were all over 40%, with hit rates of 43.1%, 41.8%, and 42.9%, respectively. The hit rate between the expert assessment and REBA assessment was 100% for the risk level 1 posture, whereas the hit rates for the risk level 2, 3, and 4 postures were 19.4 %, 1.0%, and 0%, respectively. On the work postures assessed by experts at risk level 4, REBA was all downgraded to risk level 2. For the risk level 1 posture, the hit rate of the experts’ assessment and the RULA’s assessment was 25.0%, and the risk levels 2, 3, and 4 were 76.4%, 10.2%, and 21.4%, respectively. At risk levels 1 and 2 of working postures, the hit rates between the risk assessment results of experts and the OWAS risk assessment results were 100% and 37.5%, respectively, whereas the hit rates for both the risk levels 3 and 4 were 0%. OWAS’s assessments of the working postures assessed by experts at risk levels 3 or 4 were rated at risk levels 1 or 2.

### 3.2. Quadratic Weighted Kappa Analysis 

The results of the quadratic weighted kappa analysis applied to examine the level of agreement between the assessment results of experts and existing assessment tools are shown in Table 4. Kappa’s value of experts’ evaluation and AULA’s evaluation was 0.718, which was in agreement. The kappa value between the experts’ results and the RULA, REBA, and OWAS results showed a moderate level of agreement of 0.599, 0.578, and 0.538, respectively.

### 3.3. One-Way ANOVA Analysis

**Figure 3:**
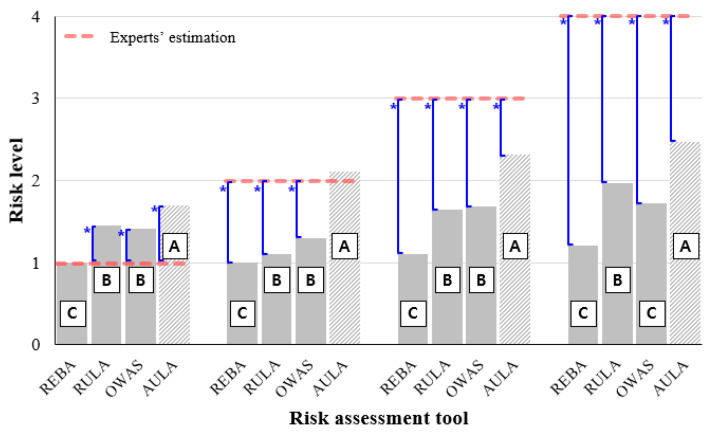
Results of four risk assessment tools and expert assessment (Note: * means significant difference in statistics; A, B, C, and D indicate significant grouping in statistics).

One-way ANOVA showed statistically significant differences between the assessment tool and the expert assessment for all four levels of risk (α < 0.05). Figure 3 shows the results of each assessment tool for each group. 

Working postures rated at risk level 1 by experts, RULA, OWAS, and AULA, showed a statistically higher risk level (over-evaluated) than the expert’s score. The result of the AULA assessment tool showed a higher risk level (1.7) than the risk levels of other tools, followed by RULA (1.45), OWAS (1.42), and REBA (1.0). For working postures rated at risk level 2 by experts, the AULA assessment results showed a level of 2.11 on average, similar to the experts’ assessments. OWAS (1.3), RULA (1.1), and REBA (1.0) analyses showed statistically significant differences from the expert assessment results. The other assessment tools except AULA showed undervaluation for working postures rated as a risk level 2. The results of the assessment tool for working postures assessed by experts at risk levels 3 and 4 also showed a trend similar to those for risk levels 1 and 2. Although all four assessment tools generally underestimated working postures rated at risk levels 3 or 4, AULA showed better results than other tools (i.e., 2.32 and 2.47 for levels 3 and 4, respectively). RULA (1.65 and 1.97), OWAS (1.69 and 1.73), and REBA (1.11 and 1.21) significantly underestimated at high risk working postures (risk levels 3 and 4).

Therefore, although AULA’s assessment of the risk level 1 working postures was slightly overrated than other tools, overall, AULA would be a useful tool for assessing different levels of work postures based on this study.

## 4. Discussion

This study was conducted to validate the AULA checklist developed for evaluating various working postures at industrial sites and agricultural sites through comparative analysis with existing ergonomic risk assessment tools (RULA, REBA, and OWAS).

As a result of the hit rate analysis, AULA had a hit rate of 41.8%–66.7% (average 48.6%), whereas RULA, developed for the analysis of upper-limb body postures, showed a hit rate of 10.2%–76.4% (average: 33.3%) in comparison with the expert’s assessment. OWAS (average: 34.4%) had a 100% hit rate for risk level 1 working postures determined by experts, but 37.5%, 0%, and 0% for risk levels 2, 3, and 4, respectively. REBA also showed similar results to OWAS, with all postures assessed at risk levels 1 and 2. Based on this analysis, REBA and OWAS generally underestimated the high-risk upper limb posture, whereas AULA and RULA, with checklists focused on upper limb posture assessment, might reflect the results of the expert assessment. In the study of Lee, Jeong, and Choe [15], REBA showed the highest correlations between perceived discomfort and assessment tool, followed by OWAS and then RULA [15]. The results showed that REBA and OWAS were developed to evaluate whole-body postures more than RULA. According to a comparative analysis of each assessment tool on lower limb postures by Kong, Lee, Lee, and Kim [26], the hit rate of ALLA, developed for lower limb posture analysis in agricultural work, was the highest compared with other checklists (RULA, REBA, and OWAS) [26]. 

Thus, we estimated that more accurate risk analysis results will be produced when appropriate assessment tools are applied according to the body parts and working position to be evaluated. In this study, we found that AULA and RULA, which focus more on upper limb posture analysis, showed a higher hit rate than REBA and OWAS, which were developed for whole-body posture analysis.

As a result of reviewing the agreement between the analysis of each assessment tool and the analysis of experts by applying the quadratic weighted kappa analysis, the kappa value (κ) of AULA was 0.718, which is relatively higher than those of other assessment tools. This means that AULA’s evaluation results well-matched those of experts. 

As a result of a one-way ANOVA on the working posture assessment tool, AULA showed the least difference from experts’ assessment of agricultural working postures at risk levels 2, 3, and 4, except for working postures at risk level 1. In this study, most assessment tools (RULA, OWAS, and AULA) showed excessive assessment of agricultural working postures assessed as risk level 1 by a group of experts [26]. For working postures with risk level 2, RULA, REBA, and OWAS presented an underestimation compared with experts’ assessment, whereas the AULA assessment was well-aligned with the expert group’s results. For risk levels 3 and 4, the overall assessment tools were rated at relatively lower risk levels than expert assessment results, but the AULA’s assessment results showed the smallest difference, followed by RULA, OWAS, and REBA in that order.

As a result of previous comparative analysis studies of assessment tools [20,21,22,23,24,29,30,31], the evaluation results of REBA and OWAS were similar to those of this study, which underestimated the risk of working posture than RULA. To prevent WMSDs and improve working postures, RULA is recommended because a more conservative assessment of working posture is desirable from a safety perspective [3,31,32,33]. 

In summary, all the assessment tools tended to under-estimate the risk level of working posture that scored higher than two points of risk level by experts. Especially at the highest risk of posture (level 4), the difference in score between the assessment tools and experts’ results was relatively significant. This implies that those assessment tools do not sufficiently reflect the risk when assessing high-risk-level working postures. As the results of the posture assessment tools are used as an index to determine whether the workplace is improved or not, under-estimation for high-risk posture should be addressed. 

For AULA, out of 14 upper limb postures, only one posture (B0-S120-E0, Figure 2) was rated level 4, which is considered the main reason for the under-estimation high-risk postures. To improve these problems, AULA should be modified using various methods such as 3D SSPP (Static Strength Prediction Program) through further studies. 

This study’s results showed that the risk assessment using AULA for agricultural working postures was relatively well-matched with the subjective risk assessment by experts compared to other evaluation tools. Thus, AULA might be a useful evaluation tool for evaluating upper extremity postures to enhance the prevention and management of WMSDs in the agricultural field. Moreover, AULA can assess the working posture relatively easily and quickly compared to the existing evaluation tools, which can reduce cost and provide time savings when investigating the hazards in the workplace.

Although AULA performed better than other evaluation tools in this study, more verification studies are required with many different working postures. AULA assessment tool is an upper limb assessment tool, so it cannot be used to assess the whole body’s risk. Thus, AULA, an evaluation tool for the upper limb, needs to be integrated with ALLA, an evaluation tool for the lower limb, and a tool for evaluating whole body posture.

## 5. Conclusions

We aimed to validate AULA against the subjective evaluation of experts and existing assessment tools (RULA, REBA, and OWAS). Both the hit rate analysis and the quadratic weighted kappa analysis showed that AULA is relatively well-aligned with experts’ subjective evaluation compared with other tools. Therefore, the AULA assessment tool and other evaluation tools might also be a useful evaluation tool for assessing working postures in agricultural sectors.

The under-estimation of AULA should be revised by using various simulation methods such as 3D-SSPP or AnyBody simulation program in further research. Despite the frequent handling of a heavyweight in the agricultural area, AULA has a limitation that weight cannot be considered a risk factor. Therefore, this limitation will also be addressed in further studies.

## Figures and Tables

**Table 1:** 10 selected crops based on the working height.

Working Height	Crop
Above shoulder	Grape, Peach, Tangerine, Pear
Near waist	Rice, Chrysanthemum
Under knee	Strawberry, Cucumber, Tomato, Oriental Melon

**Table 2:** Criteria of kappa (κ) analysis.

Kappa (*κ*)	Strength of Agreement
<0.20	Poor
0.21–0.40	Fair
0.41–0.60	Moderate
0.61–0.80	Good
>0.80	Very good

**Table 3:** Hit rates of ergonomic risk assessment tools and expert assessments [unit: %].

Experts’ Assessment	AULA				REBA				RULA				OWAS			
	1	2	3	4	1	2	3	4	1	2	3	4	1	2	3	4
1	**66.7**	33.3	-	-	**100**	-	-	-	**25.0**	75.0	-	-	**100**	-	-	-
2	33.3	**43.1**	23.6	-	80.6	**19.4**	-	-	9.7	**76.4**	13.9	-	62.5	**37.5**	-	-
3	2.0	54.1	**41.8**	2.0	29.6	69.4	**1.0**	-	40.8	49.0	**10.2**	-	20.4	79.6	-	-
4	-	42.9	14.3	**42.9**	-	100	-	-	-	7.1	71.4	**21.4**	35.7	64.3	-	-

The bold number means correct hit rate; AULA: Agricultural upper limb assessment, REBA: rapid entire body assessment, RULA: Rapid upper limb assessment, OWAS: Ovako working posture analysis system.

**Table 4:** Quadratic weighted kappa analysis of all ergonomic risk evaluation tools.

	AULA	RULA	REBA	OWAS
Kappa (*κ*)	0.718	0.599	0.578	0.538
Strength of agreement	Good	Moderate	Moderate	Moderate
